# Heme as a versatile signaling molecule: Coordinating mitochondrial metabolism with global cellular responses

**DOI:** 10.1016/j.jbc.2026.113518

**Published:** 2026-09-07

**Authors:** Iva Chitrakar, Breann L. Brown

**Affiliations:** 1 Department of Biochemistry, Vanderbilt University School of Medicine, Nashville, Tennessee, USA; 2 Center for Structural Biology, Vanderbilt University School of Medicine, Nashville, Tennessee, USA

**Keywords:** heme, mitochondria, unfolded protein response, porphyrin, iron, oxidative stress

## Abstract

Heme is an essential iron-containing cofactor that supports diverse biological processes, including oxygen transport, mitochondrial respiration, and xenobiotic metabolism. Beyond these canonical functions, accumulating evidence has established heme as a dynamic signaling molecule that couples mitochondrial metabolism to cellular stress responses, gene expression, and metabolic adaptation. Heme biosynthesis is compartmentalized between mitochondria and the cytosol, requiring tightly coordinated synthesis, trafficking, sensing, and degradation to maintain cellular homeostasis and prevent heme toxicity. In this review, we examine mechanisms by which heme regulates mitochondrial protein quality control, respiratory chain assembly, and metabolic feedback to integrate organellar function with cellular energy demands. We further discuss how heme is trafficked to extramitochondrial compartments, where it modulates cytoplasmic stress signaling, iron homeostasis, transcriptional networks, and metabolic programs through interactions with proteins, including the BACH1 transcription factor, REV-ERB nuclear receptors, and the glycolytic enzyme GAPDH. We also highlight the ongoing discourse surrounding mitochondrial heme trafficking and identify critical unanswered questions regarding the identity of intracellular heme chaperones and mitochondrial heme sensors. Finally, we discuss how dysregulation of heme metabolism contributes to diverse pathologies. Collectively, recent advances establish heme as a central regulator of mitochondrial communication and cellular homeostasis, underscoring the therapeutic potential of targeting heme signaling pathways in human disease.

Heme mediates a variety of cellular processes in many organisms, from bacteria to vertebrates. In mammals, heme is widely known for its role in oxygen transport in red blood cells, serving as the prosthetic group of globin to form hemoglobin. In addition to this ubiquitous function, heme plays other roles in maintaining cellular homeostasis. For example, heme mediates drug metabolism, serving as a fixed prosthetic group of hepatic cytochrome P450 enzymes (1). Reversible heme binding to proteins such as the circadian regulators CLOCK and the REV-ERBα nuclear receptor (2, 3, 4), as well as the transcription factor BACH1, can determine protein activity, stability, or localization (5). We refer to heme signaling as the dynamic interaction between heme and various biomolecular targets that conveys information about cellular metabolic status. This review curates myriad heme–protein interactions to highlight how heme acts as a cellular chameleon, activating and regulating several biological programs.

The heme molecule consists of a porphyrin scaffold that coordinates an iron ion in the center. In non-plant eukaryotes, heme synthesis begins with the production of aminolevulinic acid by the enzyme aminolevulinic acid synthase (ALAS) in the mitochondrial matrix (Fig. 1) (6, 7). The next four enzymatic steps occur in the cytoplasm, ensuring proper porphyrin condensation and cyclization. The final three reactions occur back inside the mitochondrion, ultimately terminating with the insertion of iron into protoporphyrin IX to form mature heme by Ferrochelatase (FECH) (8, 9, 10). Heme is then shuttled to other parts of the cell, where it performs various functions before being degraded by heme oxygenase (HO) (11, 12). Importantly, heme-mediated signal transduction extends outside of mitochondria. Therefore, it is imperative to study the role of heme in the context of its interrelated functions in cellular biology.

**Figure 1:**
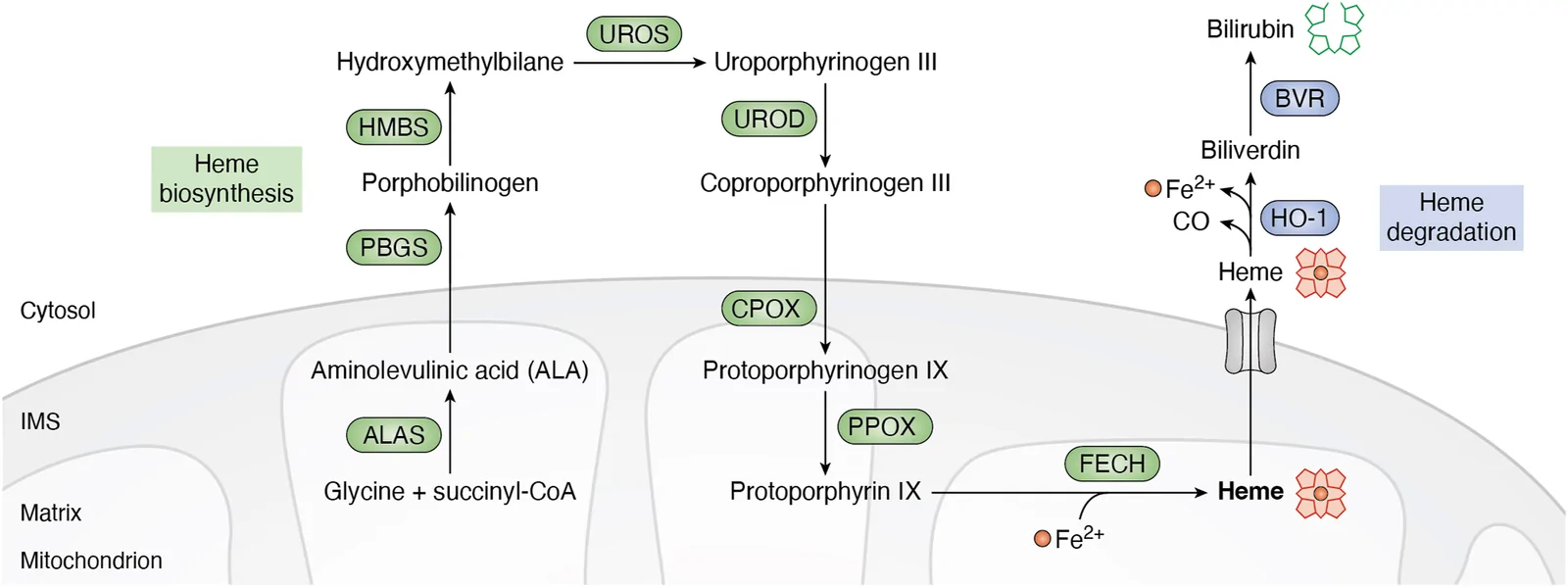
**Cellular localization of eukaryotic heme biosynthesis and degradation.** Heme synthesis is compartmentalized, beginning and terminating in the mitochondrion, with intermediate steps occurring in the cytosol. This pathway is initiated with the condensation of glycine and succinyl-CoA to form aminolevulinic acid by the enzyme Aminolevulinic Acid Synthase (ALAS). The final step requires the insertion of iron (Fe^2+^, *red circle*) into protoporphyrin IX by Ferrochelatase (FECH) to make heme (*red polygons*). Heme is then transported out of the mitochondrion (*grey channel*), where it can signal to other parts of the cell. Ultimately, Heme oxygenase 1 (HO-1) degrades free heme into carbon monoxide (CO), Fe^2+^, and biliverdin/bilirubin in the cytosol, spatially dividing heme production and catabolism. The eight enzymes catalyzing individual synthesis reactions are shown as *green ovals*. CPOX: Coproporphyrinogen-III Oxidase; HMBS: Hydroxymethylbilane synthase; PBGS, Porphobilinogen synthase; PPOX: Protoporphyrinogen Oxidase; UROD: Uroporphyrinogen Decarboxylase; UROS: Uroporphyrinogen-III Synthase. Enzymes involved in heme degradation are represented as *blue ovals*. BVR: Biliverdin Reductase. The inner and outer mitochondrial membranes are shown as *gray lines* encompassing the intermembrane space (IMS).

While this cofactor is indispensable, heme overload can lead to inflammation, organ injury, and death (13). Therefore, heme availability is tightly buffered in the cell through constant binding and exchange *via* hemoproteins and the labile heme pool. Its shuttling between subcellular compartments enables heme to link the metabolic status of the mitochondrion, cytoplasm, and nucleus, influencing multiple biological pathways. Thus, our increasing knowledge of heme-mediated signaling is revealing new and intricate links between this molecule and many areas of human physiology. We first examine mitochondrial heme signaling before discussing how heme is trafficked to extramitochondrial compartments, where it regulates cytoplasmic stress responses, nuclear gene expression, metabolism, and ultimately human disease. Understanding these regulatory mechanisms offers promising targets for therapeutic intervention in diseases ranging from anemia and neurodegeneration to cancer and inflammatory disorders.

## Mitochondrial heme signaling

Heme is not only synthesized in mitochondria but also required for proper electron transport and mitochondrial physiology. However, these organelles lack an internal mechanism to degrade heme. The absence of heme oxygenase mandates tight regulation of heme synthesis, hemoprotein maturation, and export to prevent toxic heme accumulation. Consequently, heme signaling is intricately integrated into several mitochondrial processes to regulate heme synthesis and initiate stress response when necessary (Fig. 2).

**Figure 2:**
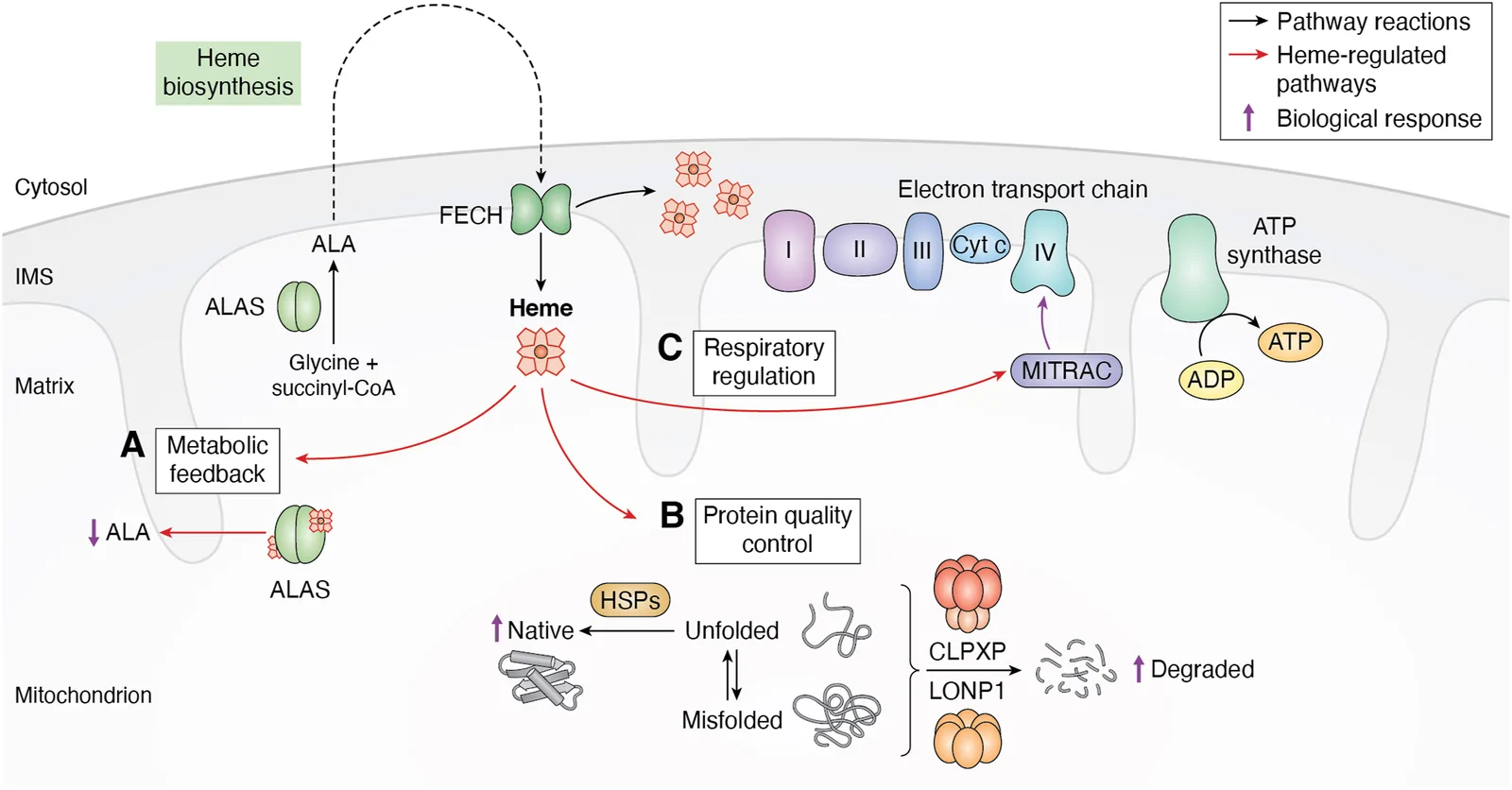
**Mitochondrial heme signaling impacts proteostasis, respiration, and metabolic feedback.** Heme availability regulates multiple major mitochondrial pathways. *A*, heme modulates its own biosynthesis through a negative feedback loop that decreases ALAS enzymatic activity and ALA production in erythroid cells, impacting the overall heme pool. *B*, proteins imported into mitochondria are subjected to protein quality control (*grey cartoons*). Maintaining proteostasis requires tight coordination between proper folding and proteolysis *via* chaperones like heat shock proteins (HSPs) and proteases (CLPXP, LONP1). In response to the heme-induced mitochondrial unfolded protein response (UPR^mt^), the chaperones and proteases operate to minimize accumulation of misfolded proteins. *C*, in parallel, heme availability coordinates electron transport chain assembly through factors such as the MITRAC complex (mitochondrial translation regulation assembly intermediate of cytochrome c oxidase), ensuring respiratory complex maturation and stabilization for efficient oxidative phosphorylation. Crosstalk between these mechanisms promotes mitochondrial metabolic homeostasis. *Black arrows* indicate pathway reactions, *red arrows* denote heme-regulated pathways, and purple arrows represent biological responses. The inner and outer mitochondrial membranes are shown as *grey lines* encompassing the intermembrane space (IMS). Heme is denoted by *red polygons*. CLPX, caseinolytic mitochondrial matrix peptidase chaperone subunit X.

### Protein quality control

Because mitochondrial function depends on the import and maturation of more than 1500 proteins, maintaining protein quality control (PQC) is essential for both respiratory function and heme homeostasis (14). The overwhelming majority of mitochondrial proteins are nuclear-encoded and must be imported into the mitochondrion either co-translationally or post-translationally (15). Proper protein import, processing, and folding are ensured by the mitochondrial PQC network (16, 17, 18, 19, 20, 21). In many instances, ATP-dependent HSPA9 and translocase of the inner mitochondrial membrane 23 (TIM23) translocase transfer nascent polypeptides into the matrix while shielding their hydrophobic regions to prevent aggregation (22, 23). Generally, once inside the mitochondrial matrix, the targeting sequence is removed, and the imported protein undergoes chaperone-mediated folding (24, 25). This network also supports proper metalation and iron–sulfur (Fe-S) cluster biogenesis by transferring the ISCU Fe-S cluster assembly scaffold to glutaredoxin-5, thereby sustaining enzymes such as FECH and respiratory chain complexes (26, 27, 28). When proteins fail to achieve their native conformation, specialized proteases recognize and degrade the misfolded polypeptides (29).

Loss of PQC can induce the mitochondrial unfolded protein response (UPR^mt^), which is a stress-response network that activates the transcription and import of nuclear-encoded mitochondrial chaperones to support proteostasis (30, 31, 32). This stress response includes the upregulation of several mitochondrial PQC players, such as the HSPA9 and HSP60 chaperones and the CLPP, LONP1, and YME1L proteases, which work in concert to refold or remove unfolded and damaged mitochondrial proteins (18, 21, 33, 34, 35, 36, 37, 38, 39). Accordingly, changes in heme levels affect mitochondrial chaperone and protease activities *via* UPR^mt^ activation. Elevated heme concentrations activate stress-responsive transcription factors, upregulating HSPA9 and HSP60 expression to alleviate proteotoxic stress (40, 41, 42, 43, 44). Conversely, heme deficiency can directly disrupt heme-binding chaperones in the mitochondrial inner membrane that are critical for the import and stability of certain electron transport chain proteins (45). The resulting accumulation of these misfolded proteins also activates the UPR^mt^ (46). Thus, mitochondrial proteostasis is unsustainable when heme pools fluctuate significantly. Interestingly, despite the swift response of mitochondrial heme levels to maintain PQC, direct heme binding to soluble chaperones in the mitochondrial matrix is yet to be identified.

A more direct mechanism linking heme signaling to PQC is the heme-dependent proteolytic degradation of ALAS. In mammals, two isoforms of ALAS exist (47, 48, 49, 50, 51, 52, 53). ALAS1 is ubiquitously expressed to support basal heme production in most tissues. ALAS2 is an erythroid-specific isoform responsible for heme synthesis needed to produce hemoglobin during red blood cell differentiation. Both isoforms possess multiple heme regulatory motifs (HRMs), composed of cysteine-proline signatures, that mediate reversible heme binding. One outcome of this interaction is the proteolytic degradation of ALAS, thereby decreasing heme production. Heme binding to an N-terminal HRM in ALAS1 recruits the ATP-dependent proteases LONP1 and CLPXP, leading to ALAS1 degradation (54, 55). Similarly, early studies suggest that heme-induced degradation of the ALAS2 isoform is mediated by CLPXP (56). This heme-dependent degradation of both ALAS isoforms establishes a negative feedback loop that prevents excess heme accumulation when its cellular levels are sufficient. Together, these mechanisms position heme as both a substrate and a regulator of mitochondrial proteostasis, ensuring that protein import, folding, and heme biosynthesis remain tightly coordinated.

### Respiratory chain assembly

Despite the fact that mitochondrial PQC preserves proteome integrity, successful protein folding alone is insufficient. Many mitochondrial proteins also require heme incorporation, making respiratory chain assembly another major point at which heme regulates mitochondrial homeostasis. Post-translational heme insertion into Complex II, Complex III, and Complex IV functions as a critical structural checkpoint that determines whether newly synthesized respiratory complexes are stable or targeted for degradation upon failure to fold properly (57, 58, 59, 60, 61). In this way, heme availability synchronizes mitochondrial translation with respiratory complex assembly, ensuring that synthesis of electron transport chain components proceeds only when cofactors are available and preventing toxic accumulation of orphan proteins (62, 63). This synchronization is especially evident during the biogenesis of Complex IV or cytochrome c oxidase, which requires the heme-binding chaperone mitochondrial translation regulation assembly intermediate of cytochrome c oxidase (MITRAC) complex to orchestrate translation with cofactor insertion (64). MITRAC assembly factors such as SURF1 and COA2 promote Complex IV maturation by facilitating proper heme insertion and stabilizing assembly intermediates (65). Notably, emerging evidence indicates that mitochondrial heme-binding assembly factors, including COA7, participate directly in Complex IV biogenesis (66). Through this proteostatic control, heme contributes to respiratory chain assembly and efficient oxidative phosphorylation.

An intriguing possibility is that this coordination is reinforced by the spatial organization of the respiratory complex assembly within a mitochondrial metabolon, a transient multi-protein complex in which enzymes and assembly factors associate to channel reactive intermediates directly between active sites (67, 68). The heme biosynthetic metabolon has been proposed to include FECH, along with inner-membrane translocases and scaffold proteins, raising the possibility that it could transiently interact with Complex IV assembly factors such as COX10, COX15, SURF1, or COA2 to facilitate the efficient delivery of newly synthesized heme to nascent respiratory complexes. Although direct evidence for such a supercomplex is lacking, this arrangement would be energetically favorable, preventing diffusion of heme and toxic precursors, limiting oxidative damage, and ensuring that heme insertion occurs only when apoproteins are properly folded and ready for maturation. Thus, heme availability not only supports respiratory complex maturation but also couples electron transport chain biogenesis to mitochondrial metabolic state, establishing the basis for broader metabolic feedback.

### Metabolic feedback

Beyond supporting respiratory complex maturation, heme synthesis is itself integrated with central carbon metabolism, potentially allowing mitochondrial metabolic flux to regulate heme production and *vice versa*. The first committed step of heme biosynthesis requires succinyl-CoA, a TCA cycle intermediate, as a precursor. ALAS condenses glycine and succinyl-CoA to produce ALA in the mitochondrion. Succinyl-CoA is generated primarily by the oxidative decarboxylation of α-ketoglutarate by the α-ketoglutarate dehydrogenase complex and can also arise from propionyl-CoA metabolism *via* the methylmalonyl-CoA pathway. In addition, the reversible succinyl-CoA synthetase reaction can interconvert succinyl-CoA and succinate, however, the physiological direction in the TCA cycle is typically toward succinate (69). Prior studies implicated multiple TCA cycle enzymes in binding to ALAS2, presumably delivering the required succinyl-CoA precursor (70, 71, 72). Speculatively, elevated ALAS activity may lower mitochondrial succinyl-CoA levels, limiting NADH and FADH_2_ generation, and directly linking heme synthesis to respiratory output and mitochondrial redox balance.

At the same time, heme itself serves as a signaling molecule to regulate its own production. For example, heme binding to the HRMs present in both ALAS1 and ALAS2 mitochondrial targeting sequences blocks mitochondrial import, providing rapid post-translational control to prevent toxicity (73, 74). Beyond this mechanism, we recently reported that within the matrix, heme acts as a reversible inhibitor by transiently binding allosteric sites on mature ALAS2, potentially stabilizing an inactive enzyme conformation (75). These mechanisms establish a local feedback system in which heme synthesis and availability influences metabolic flux. Furthermore, heme’s regulatory functions extend well beyond the organelle.

## Outer-mitochondrial heme signaling

After export from mitochondria, heme coordinates cellular adaptation by regulating cytoplasmic stress responses, nuclear transcription, and systemic metabolism (Fig. 3). Because heme is hydrophobic and cytotoxic at high concentrations, signaling depends on dedicated trafficking pathways to prevent nonspecific membrane insertion and limit oxidative damage. To date, several mechanisms for heme export and trafficking have been proposed and subsequently challenged, revealing the field’s controversial and still underdeveloped understanding.

**Figure 3:**
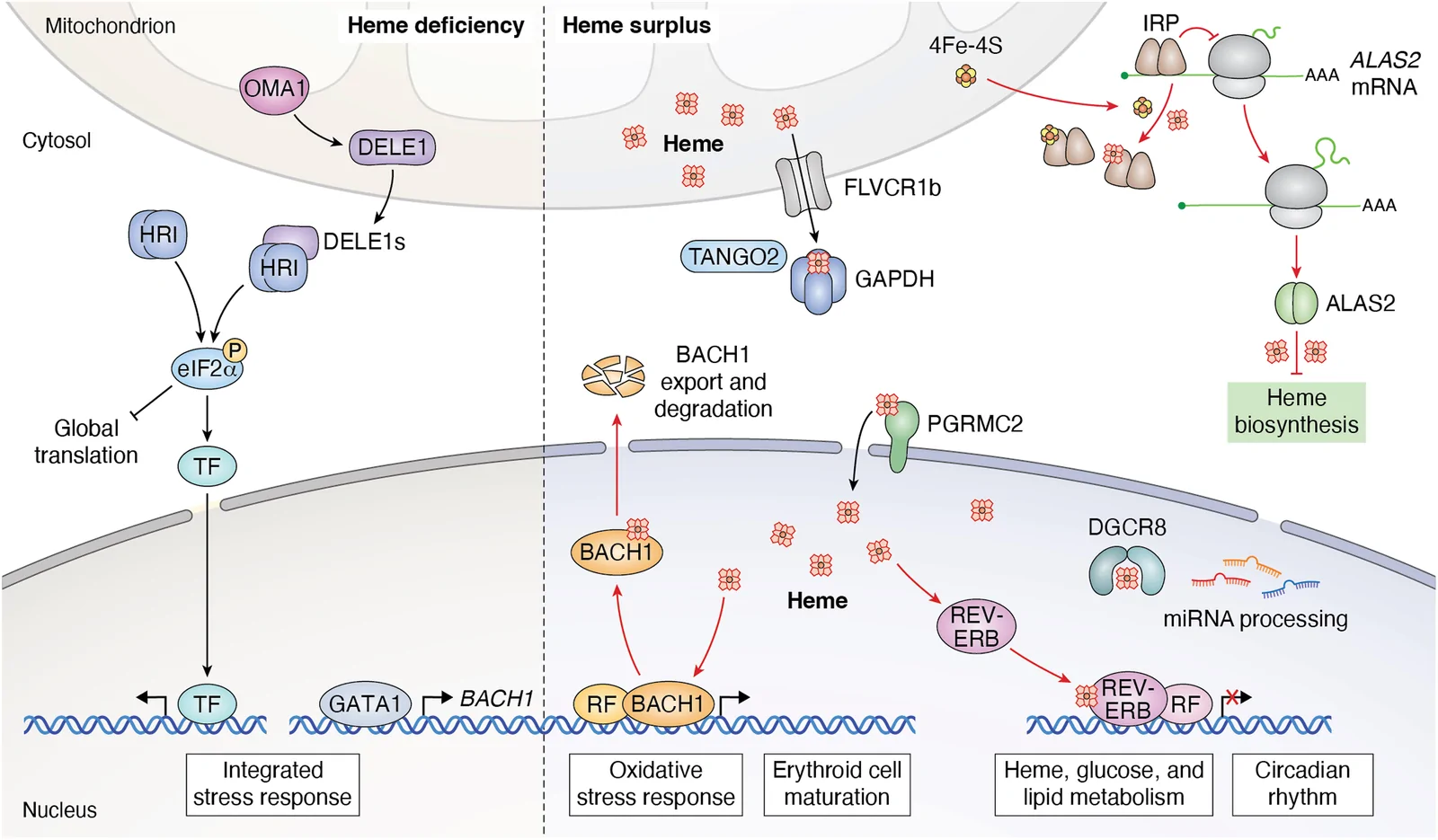
**Extramitochondrial heme signaling coordinates stress responseand gene expression.** Heme synthesized in the mitochondrion is exported to the cytosol, potentially by the mitochondrial membrane protein FLVCR1b, which coordinates with TANGO2 to transfer heme to the cytosolic chaperone GAPDH for heme distribution. Recent reports question the biological impact of this heme transfer axis since each protein also carries out other cellular functions (see main text). During heme deficiency (*left of dotted line*), two parallel pathways converge on the integrated stress response. Mitochondrial dysfunction activates the OMA1–DELE1-HRI relay, whereby OMA1 cleaves DELE1, releasing a truncated fragment (DELE1s) that directly activates the kinase HRI. Low cytosolic heme levels further relieve heme-mediated HRI inhibition. Activated HRI phosphorylates eIF2α to suppress global translation while selectively inducing stress-responsive transcription factors (TF) to restore heme balance. The master transcription factor GATA1 sustains expression of hemoglobin biosynthetic genes including *ALAS2* and *BACH1* during erythroid maturation (*black arrows*). *BACH1* transcription effectively represses heme-sensitive erythroid gene expression. Under heme surplus (*right of dotted line*), excess cytosolic heme is shuttled to the nucleus *via* the chaperone PGRMC2. Nuclear heme binds and displaces the transcription repressor BACH1 from antioxidant response elements, triggering its proteolytic degradation in the cytosol. This relieves BACH1-mediated suppression of GATA1 function, amplifying erythroid gene expression and antioxidant genes, including heme oxygenase-1, to limit oxidative damage. Additionally, loss of mitochondrial iron-sulfur clusters (4Fe-4S) converts the iron regulatory protein (IRP) from a cytosolic aconitase to a translational repressor that blocks *ALAS2* mRNA translation, suppressing heme biosynthesis. Excess heme also binds directly to ALAS, blocking mitochondrial import. Heme-bound REV-ERB nuclear receptors, in combination with various regulatory factors (RF), simultaneously repress genes governing glucose and lipid metabolism and circadian rhythm (*black arrow with red X*), forming a feedback loop that synchronizes mitochondrial heme and energy metabolism with nuclear gene expression. A final heme nuclear signaling mechanism involves microRNA (miRNA) processing *via* activation of the RNA-binding protein DiGeorge critical region-8 (DGCR8). Proteins are shown as colored shapes, and heme is depicted as *red polygons*. ALAS2: erythroid-specific aminolevulinic acid synthase; BACH1, BTB and CNC homology 1; DELE1, DAP3 Binding Cell Death Enhancer 1; eIF2α, eukaryotic translation initiation factor 2 alpha; GATA1, GATA Binding Transcription Factor; HRI, Heme-regulated eIF2α kinase; OMA1, Overlapping activity with m-AAA protease 1; PGRMC2, Progesterone Receptor Membrane Component 2; REV-ERB, reverse strand of *ERBA*.

Seminal studies identified the membrane transporter Feline Leukemia Virus subgroup C Receptor 1b (FLVCR1b) as a mitochondrial heme exporter required for erythroid differentiation, as depletion of FLVCR1b resulted in mitochondrial heme accumulation and impaired erythropoiesis (76, 77, 78). Subsequent work identified the protein transport and Golgi organization 2 (TANGO2) as a putative heme chaperone that facilitates heme transfer from mitochondria to the cytosol (79). A bacterial TANGO2 homolog was likewise shown to support cytochrome c biosynthesis and catalase activity, suggesting that a heme-binding function may be evolutionarily conserved (80). Together with later studies demonstrating heme transfer from FLVCR1b to the cytosolic chaperone GAPDH, these findings support a model in which FLVCR1b, TANGO2, and GAPDH function sequentially to export, buffer, and deliver newly synthesized heme to downstream hemoproteins and signaling targets (76, 81, 82, 83, 84, 85, 86, 87, 88).

More recent studies, however, have challenged aspects of this model. (89). Structural and functional analyses indicate that FLVCR1 and FLVCR2 transport choline and ethanolamine across the plasma membrane rather than heme (90, 91, 92, 93). Likewise, several reports suggest that many phenotypes associated with TANGO2 deficiency arise from defects in coenzyme A, lipid, or mitochondrial metabolism rather than impaired heme trafficking (89). TANGO2 is implicated in binding both the small heat shock protein crystallin alpha B and acyl-coenzyme A (94, 95) and may also play a critical role in vitamin B5 metabolism (96). These observations raise the possibility that previously observed alterations in heme homeostasis may represent indirect consequences of broader metabolic dysfunction rather than defective heme transport itself.

Rather than being mutually exclusive, these findings may reflect the complexity of intracellular heme homeostasis. FLVCR1b and TANGO2 could possess context-dependent or tissue-specific functions, participate indirectly in heme allocation by regulating mitochondrial metabolism, or act as multifunctional proteins whose roles in heme signaling vary across developmental or physiological states. Alternatively, heme export may involve multiple partially redundant pathways that compensate for one another under different conditions. Resolving these possibilities will require direct measurements of heme flux *in vivo* together with structural and biochemical characterization of candidate transporters under physiological conditions.

Additional proteins have also been implicated in intracellular heme trafficking. The membrane-associated protein PGRMC1 localizes to mitochondria–ER contact sites and interacts with several heme biosynthetic enzymes, suggesting a role in coordinating heme transfer between organelles (97, 98). PGRMC1 also associates with PGRMC2, which has been proposed to facilitate nuclear heme delivery for transcriptional regulation (99). Collectively, these studies support the existence of a highly regulated intracellular trafficking network, although the identities, mechanisms, and relative contributions of its individual components remain active areas of investigation.

### Cytoplasmic stress response

Cells rapidly adapt to mitochondrial dysfunction by reprogramming protein synthesis through the integrated stress response (ISR) (100). These changes in translation are mediated by the phosphorylation of eukaryotic translation initiation factor 2 alpha (eIF2α) by one of four eIF2α kinases, including the Heme-regulated eIF2α kinase (HRI). HRI receives signals directly from stressed mitochondria through the OMA1-DELE-HRI relay (20, 21, 38, 101, 102). OMA1 is an inner mitochondrial membrane metalloprotease that is activated under conditions that compromise mitochondrial PQC, such as arrested protein import, accumulation of misfolded proteins, loss of membrane potential, or defects in mitochondrial metabolism. Upon activation, OMA1 cleaves the mitochondrial protein DELE1 (DAR2-binding cell death enhancer 1). Cytosolic accumulation of processed DELE1 directly activates HRI, prompting ISR signaling even when cytosolic heme levels are sufficient (103). This pathway enables mitochondrial dysfunction and iron deficiency to rapidly reprogram cytoplasmic protein synthesis. In parallel, HRI activity is inhibited when heme is bound to the N-terminal domain or the kinase insertion domain (104, 105). This inhibition is reversed in response to low cytosolic heme levels or by HSP70 sequestration of misfolded or aggregated proteins during proteotoxic stress. Both effectively release HRI, which phosphorylates eIF2α to halt global protein synthesis while also inducing translation of stress-responsive transcription factors to adapt to oxidative and proteotoxic stress (106).

Iron and heme availability operate in parallel with HRI, providing an additional layer of regulation. This adaptation is fulfilled by iron regulatory proteins (IRP1 and IRP2), bifunctional cytosolic RNA-binding proteins that post-transcriptionally regulate cellular iron metabolism. Under basal iron conditions, IRP functions as cytosolic aconitase (107, 108). During mitochondrial dysfunction or iron deficiency, the heme-IRP switch prioritizes cellular survival over heme and hemoglobin production (109). Loss of its 4Fe-4S cluster converts IRP into a translational repressor that blocks *ALAS2* translation while stabilizing transferrin receptor mRNA to increase iron uptake and prevent toxic accumulation of porphyrin intermediates (110). Heme also directly controls IRP activity by sterically blocking IRP binding to iron-responsive elements (IREs) located in the 5′ untranslated region of *ALAS2* mRNA, and by serving as a signal to recruit E3 ubiquitin ligases for subsequent IRP proteasomal degradation (111, 112, 113). One exception to this model is during terminal erythroid differentiation, where the IRP system is less responsive to cellular iron levels to ensure robust heme synthesis for hemoglobinization (114).

While IRP1 suppresses heme synthesis, HRI decreases protein synthesis to prevent heme accumulation (115). Because IRP1 activation depends on mitochondrial 4Fe-4S cluster biogenesis, it is plausible that proteotoxic stress that sequesters mitochondrial chaperones such as HSPA9 could indirectly suppress heme biosynthesis. Collectively, these pathways allow cytoplasmic translation to rapidly respond to changes in both mitochondrial function and heme availability prior to a transcriptional response.

### Nuclear gene expression

Whereas cytoplasmic heme signaling enables rapid translational adaptation, this molecule also elicits longer-lasting responses by directly regulating nuclear transcription factors and receptors. One well-characterized pathway is mediated by the transcriptional repressor BACH1, which binds antioxidant response elements and suppresses genes such as heme oxygenase-1 (HO-1) when heme is low. When heme levels rise, its binding promotes BACH1 nuclear export and proteasomal degradation (116), relieving repression of NRF2-dependent antioxidant genes (117). NRF2, normally restrained by KEAP1 and BACH1, then induces targets including HO-1, which catabolizes excess heme into biliverdin, iron, and carbon monoxide (CO) to limit oxidative damage (118, 119, 120). Thus, BACH1 degradation and NRF2-dependent activation of antioxidant and heme-degradation genes coordinate mitochondrial respiration and reactive oxygen species production with nuclear antioxidant gene expression.

In addition to stress-responsive transcription factors, heme also regulates nuclear receptors that synchronize metabolism and circadian rhythms. REV-ERB nuclear receptors (REV-ERBα/NR1D1 and REV-ERBβ/NR1D2) are ligand-dependent transcriptional repressors (4). Heme-bound REV-ERBs repress genes involved in gluconeogenesis, lipid metabolism, and circadian regulation (2, 4, 121, 122, 123). Furthermore, despite the oxidative state of heme-bound REV-ERBs, they act as gas sensors, with CO and nitric oxide (NO) modulating their transcriptional activity (124). Along with heme-binding circadian regulators, these pathways convey the mitochondrial metabolic state to the nucleus (125, 126). Accordingly, REV-ERB signaling provides a direct mechanism by which mitochondrial heme availability reshapes long-term metabolic gene expression.

Heme-nuclear communication is particularly evident in erythroid cells, where heme and globin synthesis must remain tightly synchronized to minimize pathophysiologic conditions such as Thalassemia, a blood disease in which globin production outweighs heme availability. Erythroid cells maintain this balance through a transcriptional circuit centered on GATA1, BACH1, and heme. GATA1 is the master transcription factor that drives erythroid differentiation by activating genes required for erythrocyte maturation, including those involved in heme biosynthesis (such as *ALAS2*) and hemoglobin synthesis (48, 127). Genome-wide studies have identified BACH1 as a direct or indirect component of the GATA1 transcriptional network during erythropoiesis (128, 129). BACH1 subsequently functions as a heme-sensitive transcriptional repressor of the β-globin locus and other heme-responsive genes by binding *MAF* recognition elements as a heterodimer with small MAF proteins, thereby antagonizing transcriptional activation by NRF2 (119, 120). This regulatory architecture forms a coherent feed-forward circuit in which GATA1 promotes erythroid differentiation while simultaneously inducing a transient repressor that restrains globin gene expression until intracellular heme levels are sufficient. Heme-mediated inhibition and degradation of BACH1 subsequently relieve this repression, ensuring that globin synthesis is tightly coordinated with heme availability (116). Unlike the rapid translational response mediated by HRI and IRPs, these heme-responsive transcriptional circuits provide a mechanism for sustained adaptation to changes in heme availability.

### Metabolic signaling

The transcriptional programs initiated by heme sensing ultimately converge on broader metabolic adaptations that coordinate mitochondrial activity with global cell physiology. In addition to a multitude of biological events dictated by heme-activated REV-ERB nuclear receptors, it also influences the REV-ERB/PGC-1α feedback loop. Peroxisome proliferator-activated receptor gamma coactivator 1α, PGC-1α, is a transcriptional coactivator that promotes mitochondrial biogenesis, oxidative metabolism, and adaptive energy homeostasis. REV-ERBα represses transcription of *PGC-1α* when bound to heme, while PGC-1α is also a positive regulator of hepatic *ALAS1* expression (121, 130). By responding to heme, this pathway forms a feedback loop, such that this cofactor represses its own synthesis. Through this mechanism, mitochondrial heme production is synchronized with mitochondrial biogenesis and energy metabolism. Moreover, REV-ERB/PGC-1α signaling extends to broader lipid metabolic networks. REV-ERBα also regulates the promoters of lipid transport proteins such as apolipoprotein CIII (131, 132).

Heme also controls metabolism through post-translational signaling pathways. For instance, heme activation of soluble guanylate cyclase (sGC), the primary receptor for NO in vascular signaling, is essential for converting apo-sGC into an NO-responsive holoenzyme. NO promotes heme insertion, directly activating apo-sGC, creating a circuit by which NO signaling enhances maturation of its own receptor (133). Recent theories suggest NO plays a role in heme allocation beyond sGC (134). Several hemoproteins are known to be only partially saturated with heme in the cell, which would allow for graded regulation of protein activity *via* heme signaling without requiring changes in protein expression. Since NO has a concentration-dependent effect on cell heme allocation, stimulating heme delivery at low NO concentrations, it could serve as a master regulator of heme signaling *via* altered incorporation into myriad hemoproteins (135).

Further, heme facilitates microRNA (miRNA) stability and processing, both directly and indirectly. First, heme directly binds the RNA-binding protein DiGeorge critical region-8 (DGCR8), leading to its dimerization and activation of miRNA primary transcript (pri-miRNA) processing (136). Additionally, ALAS1 was recently shown to repress cytosolic miRNA accumulation in a heme-independent manner (137). Together, these mechanisms suggest heme signaling operates across multiple regulatory layers, with changes in heme pool impacting transcription, protein activity and RNA processin.

## Heme and pathogenesis

The signaling pathways described above normally align mitochondrial function with cellular adaptation. Consequently, disruption of heme synthesis, trafficking, sensing, or degradation compromises these homeostatic networks, promoting numerous human diseases (Fig. 4). Defects in heme synthesis are known to cause several types of porphyrias, anemia, and Friedreich's ataxia. In hepatic porphyrias, insufficient enzyme activity leads to the accumulation of toxic porphyrin precursors, causing acute neurovisceral attacks characterized by severe abdominal pain and neurologic symptoms and/or cutaneous photosensitivity with blistering skin lesions (130, 138, 139). Mutations in erythroid-specific ALAS2 impair heme synthesis, leading to mitochondrial iron overload and ineffective erythropoiesis in X-linked sideroblastic anemia (140). In another example, mutations in the *FXN* gene cause nerve damage underlying Friedreich's ataxia, a rare inherited disease that affects the nerves and heart, leading to problems with balance, coordination, walking, speech, and muscle strength (141, 142). Although earlier studies reported that Frataxin directly delivers iron to FECH, recent work describes the primary role of Frataxin in Fe-S cluster biogenesis (143), which, in turn, may affect FECH stability and heme synthesis. Moreover, mitochondrial protein quality control malfunctions, such as defective cytochrome assembly due to mutations in the heme delivery protein SURF1 and cytochrome delivery factors COX10 and COX15, could destabilize respiratory complexes (144, 145, 146, 147, 148, 149). Clinically, this can lead to disorders such as Leigh syndrome and related mitochondrial encephalopathies, which are characterized by progressive damage to the brain and nervous system, causing developmental delays, muscle weakness, movement problems, seizures, and difficulty with breathing or swallowing.

**Figure 4:**
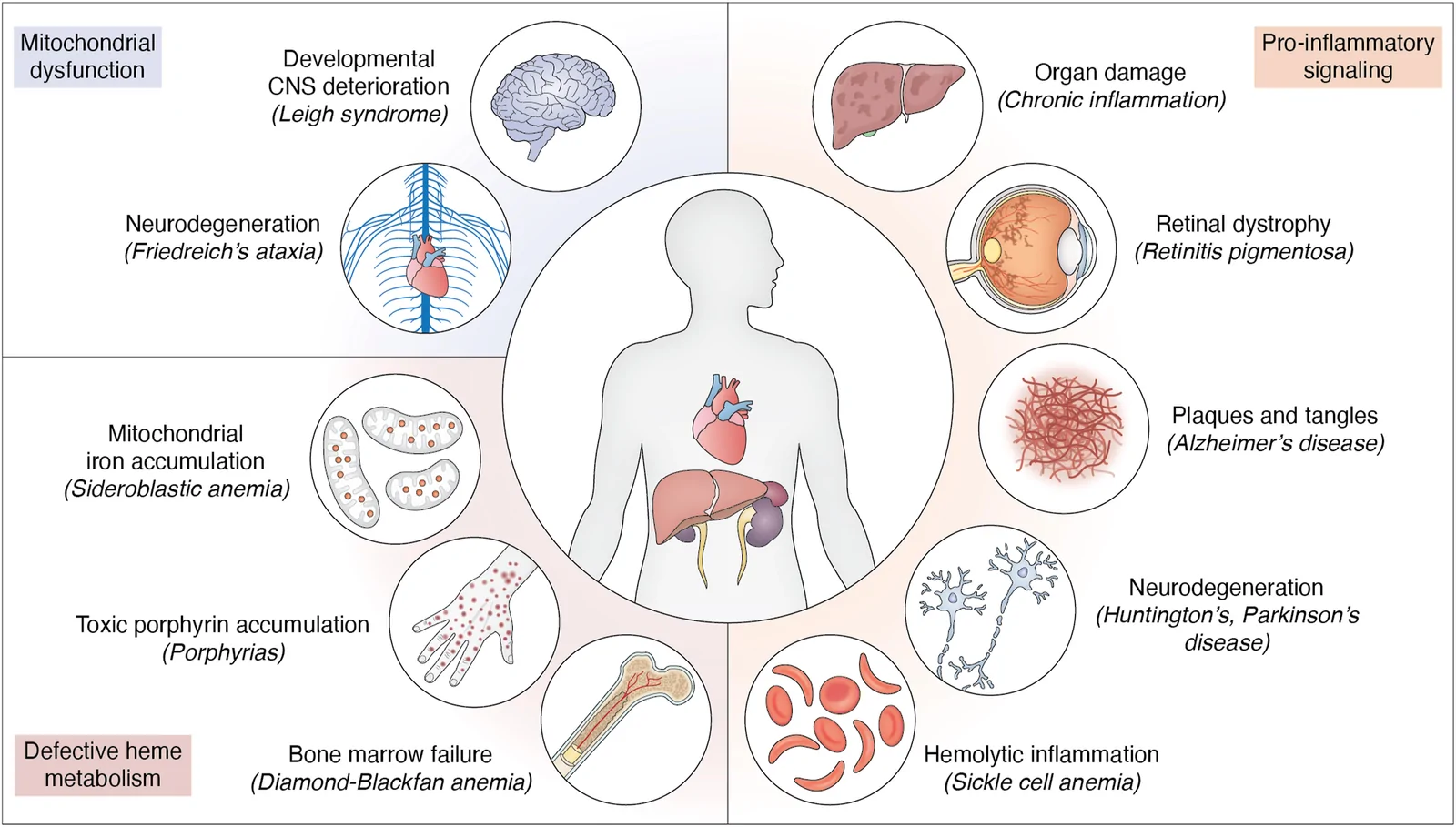
**Disruption of heme synthesis, trafficking, sensing, and degradation underlies various human diseases.** Dysregulation of heme homeostasis produces pathological consequences, here broadly grouped into mitochondrial dysfunction (*upper left*), defective heme metabolism (*lower left*), and pro-inflammatory signaling (*right*), affirming the widespread roles of heme signaling. Defective mitochondrial protein quality control destabilizes respiratory complexes, manifesting clinically as Leigh syndrome and related mitochondrial encephalopathies that affect central nervous system (CNS) development. Disruption in iron–sulfur cluster biogenesis impairs Ferrochelatase stability and heme synthesis, contributing to the neurodegeneration underlying Friedreich’s ataxia. Multiple enzymatic deficiencies in heme biosynthesis may cause toxic accumulation of porphyrin precursors, a hallmark of porphyrias. Other perturbations that impair heme synthesis drive mitochondrial iron overload, leading to sideroblastic anemia. Disruption of heme export also has severe consequences, as FLVCR1 mutations cause retinal dystrophy and bone marrow failure, but the role of aberrant heme transport in disease etiology is currently debated. Free heme can also be recognized as a damage-associated molecular pattern, activating innate immune cells and promoting chronic inflammation. Heme-induced oxidative stress and protein misfolding further promote neurodegeneration in Parkinson’s, Alzheimer’s, and Huntington’s diseases, with aberrant heme oxygenase activity and accumulation of heme degradation products generating reactive oxygen species that amplify cellular injury. When heme exceeds cellular buffering capacity, as in hemolytic disorders such as sickle cell disease, free extracellular heme activates innate immune pathways and drives vascular inflammation. Collectively, these pathologies illustrate how perturbation across heme metabolism, from biosynthesis and trafficking to sensing and degradation, converts a tightly regulated signaling molecule into an instigator of chronic diseases.

Since heme trafficking is also critical, disruption in heme export leads to numerous pathologies. Mutation of the FLVCR1 transporter impacts the nervous system, leading to various diseases, including retinitis pigmentosa (150, 151), and developmental syndromes, such as Diamond-Blackfan Anemia (152, 153). In parallel, free heme acts as a damage-associated molecular pattern leading to pro-inflammatory responses. Hemolytic disorders, such as sickle cell disease, highlight how extracellular heme can activate innate immune pathways and induce vascular inflammation when its buffering systems are overwhelmed. Oxidative stress and protein misfolding are hallmarks of multiple neurodegenerative disorders, such as Parkinson’s, Alzheimer’s, and Huntington’s diseases (154, 155). Tumor cells frequently reprogram heme biosynthesis to co-opt heme signaling to support proliferation and metastasis, altering heme transport (156, 157, 158) and transcription factors such as BACH1 and NRF2 (159, 160). Finally, aberrant heme degradation also drives pathogenesis. Prior studies report that the levels of both heme oxygenase and bilirubin increase in patients with Alzheimer’s disease (161, 162, 163). This pathological regulation also extends to the products of heme degradation, including iron and CO, which can generate reactive oxygen species or bind ion channels, altering their function (164, 165, 166, 167). Together, these disorders underscore that heme is far more than a metabolic cofactor; its synthesis, trafficking, sensing, and degradation form an integrated signaling network whose disruption factors into a broad spectrum of human disease.

## Outlook

Despite major advances in identifying heme-responsive signaling pathways, fundamental questions remain regarding how cells spatially organize, traffic, and sense heme. Addressing these questions represents the next frontier in understanding heme as a signaling molecule.

One of the most pressing challenges is identifying the proteins responsible for intracellular heme trafficking. The recent discovery that GAPDH functions as a cytosolic heme chaperone transformed our understanding of heme allocation by demonstrating that enzymes with established metabolic functions can also participate directly in heme signaling. This finding raises the possibility that additional multifunctional proteins remain undiscovered. The ongoing controversy over the identities of mitochondrial heme transporters, including FLVCR1b and TANGO2, further underscores the evolving discourse on the mechanisms governing intracellular heme transport. Likewise, no high-resolution structure of a eukaryotic heme chaperone bound to its hemoprotein target has yet been reported, leaving fundamental questions regarding substrate recognition and specificity unanswered.

These uncertainties also raise broader conceptual questions about mitochondrial heme signaling itself. Despite the mitochondrion being the exclusive site of heme synthesis, comparatively few mitochondrial heme-sensing pathways have been identified relative to those operating in the cytoplasm and nucleus. One possibility is that eukaryotic cells restrict mitochondrial heme signaling to a small number of tightly regulated processes, such as ALAS feedback inhibition and heme-dependent proteolysis, thereby minimizing fluctuations in labile heme. Alternatively, heme distribution within mitochondria may occur through a transient metabolon that channels newly synthesized heme directly between biosynthetic enzymes, assembly factors, and respiratory complexes. The proposed association of FECH with inner membrane scaffold proteins provides an attractive framework for such a mechanism (68, 168), although direct experimental evidence remains limited. An equally intriguing possibility is that mitochondrial heme sensors have escaped identification because they moonlight as proteins with established metabolic or chaperone functions, analogous to GAPDH in the cytosol. Distinguishing among these models will require identifying *bona fide* mitochondrial heme-binding proteins, determining their heme affinities and regulatory thresholds, and defining how heme is selectively retained, trafficked, or exported from the mitochondrial matrix.

Addressing these gaps will depend on continued technological innovation. Genetically encoded fluorescent heme sensors have already begun to reveal the spatial and temporal dynamics of labile heme in cells. When combined with genome-wide genetic perturbation approaches such as CRISPR interference, targeted protein degradation systems, and quantitative proteomics, these sensors should enable systematic identification of new components of the heme signaling network (88). Integrating these approaches with structural biology and quantitative measurements of compartment-specific labile heme will provide a more complete understanding of how cells regulate heme allocation under both physiological and pathological conditions.

Finally, translating these mechanistic insights into therapeutic strategies remains an important long-term goal. Cellular concentrations of labile and protein-bound heme vary substantially across tissues, developmental stages, and metabolic states, yet the thresholds that distinguish physiological signaling from toxicity remain poorly defined (62, 169, 170, 171). Because dysregulated heme signaling triggers disorders ranging from porphyrias and mitochondrial disease to neurodegeneration, inflammation, and cancer, future therapies must restore heme homeostasis without disrupting essential hemoprotein biogenesis. Achieving this balance will require a detailed understanding of when, where, and how heme functions as a signaling molecule. As the molecular framework of heme trafficking and sensing continues to emerge, targeting these pathways may provide new opportunities to selectively modulate mitochondrial function and cellular adaptation in human disease.

## Conflict of interest

The authors declare that they have no conflicts of interest with the contents of this article.

## References

[1] Guengerich F.P. (1992). Characterization of human cytochrome P450 enzymes. FASEB J..

[2] Yin L., Wu N., Curtin J.C., Qatanani M., Szwergold N.R., Reid R.A. (2007). Rev-erbalpha, a heme sensor that coordinates metabolic and circadian pathways. Science.

[3] Freeman S.L., Kwon H., Portolano N., Parkin G., Venkatraman Girija U., Basran J. (2019). Heme binding to human CLOCK affects interactions with the E-box. Proc. Natl. Acad. Sci. U. S. A..

[4] Raghuram S., Stayrook K.R., Huang P., Rogers P.M., Nosie A.K., McClure D.B. (2007). Identification of heme as the ligand for the orphan nuclear receptors REV-ERBalpha and REV-ERBbeta. Nat. Struct. Mol. Biol..

[5] Ogawa K., Sun J., Taketani S., Nakajima O., Nishitani C., Sassa S. (2001). Heme mediates derepression of Maf recognition element through direct binding to transcription repressor Bach1. EMBO J..

[6] Gibson K.D., Laver W.G., Neuberger A. (1958). Initial stages in the biosynthesis of porphyrins. 2. The formation of delta-aminolaevulic acid from glycine and succinyl-coenzyme A by particles from chicken erythrocytes. Biochem. J..

[7] Kikuchi G., Kumar A., Talmage P., Shemin D. (1958). The enzymatic synthesis of delta-aminolevulinic acid. J. Biol. Chem..

[8] Ashenbrucker H., Cartwright G.E., Goldberg A., Wintrobe M.M. (1956). Studies on the biosynthesis of heme in vitro by avian erythrocytes. Blood.

[9] Klein J.R., Krueger R.C., Melnick I. (1956). Formation of heme by broken-cell preparations of duck erythrocytes. Arch. Biochem. Biophys..

[10] Wu C.K., Dailey H.A., Rose J.P., Burden A., Sellers V.M., Wang B.C. (2001). The 2.0 A structure of human ferrochelatase, the terminal enzyme of heme biosynthesis. Nat. Struct. Biol..

[11] Maines M.D., Ibrahim N.G., Kappas A. (1977). Solubilization and partial purification of heme oxygenase from rat liver. J. Biol. Chem..

[12] Yoshida T., Kikuchi G. (1978). Features of the reaction of heme degradation catalyzed by the reconstituted microsomal heme oxygenase system. J. Biol. Chem..

[13] Voltarelli V.A., Alves de Souza R.W., Miyauchi K., Hauser C.J., Otterbein L.E. (2023). Heme: the lord of the iron ring. Antioxidants (Basel).

[14] Pagliarini D.J., Calvo S.E., Chang B., Sheth S.A., Vafai S.B., Ong S.E. (2008). A mitochondrial protein compendium elucidates complex I disease biology. Cell.

[15] Zhu Z., Mallik S., Stevens T.A., Huang R., Levy E.D., Shan S.O. (2025). Principles of cotranslational mitochondrial protein import. Cell.

[16] Chen T.H., Chou C.K., Lin K.M. (2026). Aberrant mitochondrial hsp60 expression affects mitochondria homeostasis and results in muscle dystrophy and premature death. Cell Death Dis..

[17] Szego E.M., Dominguez-Meijide A., Gerhardt E., Konig A., Koss D.J., Li, W. (2019). Cytosolic trapping of a mitochondrial heat shock protein is an early pathological event in synucleinopathies. Cell Rep..

[18] Tian G., Hu C., Yun Y., Yang W., Dubiel W., Cheng Y. (2021). Dual roles of HSP70 chaperone HSPA1 in quality control of nascent and newly synthesized proteins. EMBO J..

[19] Rajagopal A., Rao A.U., Amigo J., Tian M., Upadhyay S.K., Hall C. (2008). Haem homeostasis is regulated by the conserved and concerted functions of HRG-1 proteins. Nature.

[20] Guo X., Aviles G., Liu Y., Tian R., Unger B.A., Lin Y.T. (2020). Mitochondrial stress is relayed to the cytosol by an OMA1-DELE1-HRI pathway. Nature.

[21] Bakovic P., Mirosevic V., Svagusa T., Sepac A., Kulic A., Milicic D. (2025). Reduced expression of UPRmt proteins HSP10, HSP60, HTRA2, OMA1, SPG7, and YME1L is associated with accelerated heart failure in humans. Biomedicines.

[22] Berthold J., Bauer M.F., Schneider H.C., Klaus C., Dietmeier K., Neupert W. (1995). The MIM complex mediates preprotein translocation across the mitochondrial inner membrane and couples it to the mt-Hsp70/ATP driving system. Cell.

[23] Moro F., Sirrenberg C., Schneider H.C., Neupert W., Brunner M. (1999). The TIM17.23 preprotein translocase of mitochondria: composition and function in protein transport into the matrix. EMBO J..

[24] Neupert W., Herrmann J.M. (2007). Translocation of proteins into mitochondria. Annu. Rev. Biochem..

[25] Bottinger L., Oeljeklaus S., Guiard B., Rospert S., Warscheid B., Becker T. (2015). Mitochondrial heat shock protein (Hsp) 70 and Hsp10 cooperate in the formation of Hsp60 complexes. J. Biol. Chem..

[26] Albetel A.N., Outten C.E. (2018). Characterization of glutaredoxin Fe-S cluster-binding interactions using circular dichroism spectroscopy. Methods Enzymol..

[27] Shakamuri P., Zhang B., Johnson M.K. (2012). Monothiol glutaredoxins function in storing and transporting [Fe2S2] clusters assembled on IscU scaffold proteins. J. Am. Chem. Soc..

[28] Crooks D.R., Ghosh M.C., Haller R.G., Tong W.H., Rouault T.A. (2010). Posttranslational stability of the heme biosynthetic enzyme ferrochelatase is dependent on iron availability and intact iron-sulfur cluster assembly machinery. Blood.

[29] Tatsuta T., Langer T. (2008). Quality control of mitochondria: protection against neurodegeneration and ageing. EMBO J..

[30] Nargund A.M., Pellegrino M.W., Fiorese C.J., Baker B.M., Haynes C.M. (2012). Mitochondrial import efficiency of ATFS-1 regulates mitochondrial UPR activation. Science.

[31] Ge J., Zhang C., Sun Y.C., Zhang Q., Lv M.W., Guo K. (2019). Cadmium exposure triggers mitochondrial dysfunction and oxidative stress in chicken (Gallus gallus) kidney via mitochondrial UPR inhibition and Nrf2-mediated antioxidant defense activation. Sci. Total Environ..

[32] Papa L., Germain D. (2014). SirT3 regulates the mitochondrial unfolded protein response. Mol. Cell Biol..

[33] Brambley C.A., Marsee J.D., Halper N., Miller J.M. (2019). Characterization of mitochondrial YME1L protease oxidative stress-induced conformational state. J. Mol. Biol..

[34] MacVicar T., Ohba Y., Nolte H., Mayer F.C., Tatsuta T., Sprenger H.G. (2019). Lipid signalling drives proteolytic rewiring of mitochondria by YME1L. Nature.

[35] Cesnekova J., Rodinova M., Hansikova H., Zeman J., Stiburek L. (2018). Loss of mitochondrial AAA proteases AFG3L2 and YME1L impairs mitochondrial structure and respiratory chain biogenesis. Int. J. Mol. Sci..

[36] Ruan Y., Li H., Zhang K., Jian F., Tang J., Song Z. (2013). Loss of Yme1L perturbates mitochondrial dynamics. Cell Death Dis..

[37] Stiburek L., Cesnekova J., Kostkova O., Fornuskova D., Vinsova K., Wenchich L. (2012). YME1L controls the accumulation of respiratory chain subunits and is required for apoptotic resistance, cristae morphogenesis, and cell proliferation. Mol. Biol. Cell.

[38] Rainbolt T.K., Saunders J.M., Wiseman R.L. (2015). YME1L degradation reduces mitochondrial proteolytic capacity during oxidative stress. EMBO Rep..

[39] Simonova V.V., Guzeev M.A., Ekimova I.V., Pastukhov Y.F. (2022). Chaperone Hsp70 (HSPA1) is involved in the molecular mechanisms of sleep cycle integration. Int. J. Mol. Sci..

[40] Lin K.M., Lin B., Lian I.Y., Mestril R., Scheffler I.E., Dillmann W.H. (2001). Combined and individual mitochondrial HSP60 and HSP10 expression in cardiac myocytes protects mitochondrial function and prevents apoptotic cell deaths induced by simulated ischemia-reoxygenation. Circulation.

[41] Comert C., Kjaer-Sorensen K., Hansen J., Carlsen J., Just J., Meaney B.F. (2024). HSP60 chaperone deficiency disrupts the mitochondrial matrix proteome and dysregulates cholesterol synthesis. Mol. Metab..

[42] Cao S., Li Y., Chen L., Lei X., Feng X., Li Y. (2025). HSP60 inhibits DF-1 apoptosis through its mitochondrial signal peptide. Poult. Sci..

[43] Cabiscol E., Belli G., Tamarit J., Echave P., Herrero E., Ros J. (2002). Mitochondrial Hsp60, resistance to oxidative stress, and the labile iron pool are closely connected in Saccharomyces cerevisiae. J. Biol. Chem..

[44] Liyanagamage D., Martinus R.D. (2020). Role of mitochondrial stress protein HSP60 in diabetes-induced neuroinflammation. Mediators Inflamm..

[45] Atamna H., Liu J., Ames B.N. (2001). Heme deficiency selectively interrupts assembly of mitochondrial complex IV in human fibroblasts: revelance to aging. J. Biol. Chem..

[46] Rolland S.G., Schneid S., Schwarz M., Rackles E., Fischer C., Haeussler S. (2019). Compromised mitochondrial protein import acts as a signal for UPR(mt). Cell Rep..

[47] Srivastava G., Borthwick I.A., Maguire D.J., Elferink C.J., Bawden M.J., Mercer J.F. (1988). Regulation of 5-aminolevulinate synthase mRNA in different rat tissues. J. Biol. Chem..

[48] Surinya K.H., Cox T.C., May B.K. (1998). Identification and characterization of a conserved erythroid-specific enhancer located in intron 8 of the human 5-aminolevulinate synthase 2 gene. J. Biol. Chem..

[49] Yamamoto M., Yew N.S., Federspiel M., Dodgson J.B., Hayashi N., Engel J.D. (1985). Isolation of recombinant cDNAs encoding chicken erythroid delta-aminolevulinate synthase. Proc. Natl. Acad. Sci. U. S. A..

[50] Sutherland G.R., Baker E., Callen D.F., Hyland V.J., May B.K., Bawden M.J. (1988). 5-Aminolevulinate synthase is at 3p21 and thus not the primary defect in X-linked sideroblastic anemia. Am. J. Hum. Genet..

[51] Bishop D.F., Henderson A.S., Astrin K.H. (1990). Human delta-aminolevulinate synthase: assignment of the housekeeping gene to 3p21 and the erythroid-specific gene to the X chromosome. Genomics.

[52] Cox T.C., Bawden M.J., Abraham N.G., Bottomley S.S., May B.K., Baker E. (1990). Erythroid 5-aminolevulinate synthase is located on the X chromosome. Am. J. Hum. Genet..

[53] Cotter P.D., Willard H.F., Gorski J.L., Bishop D.F. (1992). Assignment of human erythroid delta-aminolevulinate synthase (ALAS2) to a distal subregion of band Xp11.21 by PCR analysis of somatic cell hybrids containing X; autosome translocations. Genomics.

[54] Kardon J.R., Moroco J.A., Engen J.R., Baker T.A. (2020). Mitochondrial ClpX activates an essential biosynthetic enzyme through partial unfolding. Elife.

[55] Nomura K., Kitagawa Y., Aihara M., Ohki Y., Furuyama K., Hirokawa T. (2021). Heme-dependent recognition of 5-aminolevulinate synthase by the human mitochondrial molecular chaperone ClpX. FEBS Lett..

[56] Cottle T., Joh L., Posner C., DeCosta A., Campagna D.R., Fleming M.D. (2026). An adaptor for feedback regulation of heme biosynthesis by a mitochondrial protease. Science.

[57] Soto I.C., Fontanesi F., Myers R.S., Hamel P., Barrientos A. (2012). A heme-sensing mechanism in the translational regulation of mitochondrial cytochrome c oxidase biogenesis. Cell Metab..

[58] Soto I.C., Barrientos A. (2016). Mitochondrial cytochrome c oxidase biogenesis is regulated by the Redox State of a heme-binding translational activator. Antioxid. Redox Signal..

[59] Guaras A., Perales-Clemente E., Calvo E., Acin-Perez R., Loureiro-Lopez M., Pujol C. (2016). The CoQH2/CoQ ratio serves as a sensor of respiratory chain efficiency. Cell Rep..

[60] Keilin D., Hartree E.F. (1947). Activity of the cytochrome system in heart muscle preparations. Biochem. J..

[61] McMinimy R., Manford A.G., Gee C.L., Chandrasekhar S., Mousa G.A., Chuang J. (2024). Reactive oxygen species control protein degradation at the mitochondrial import gate. Mol. Cell.

[62] Gallio A.E., Marson N.A., Heesom K.J., Lewis P.A., Alibhai D., Dugdale C.A. (2025). An extended network for regulation of heme homeostasis in cells. Proc. Natl. Acad. Sci. U. S. A..

[63] Mobius K., Arias-Cartin R., Breckau D., Hannig A.L., Riedmann K., Biedendieck R. (2010). Heme biosynthesis is coupled to electron transport chains for energy generation. Proc. Natl. Acad. Sci. U. S. A..

[64] Mick D.U., Dennerlein S., Wiese H., Reinhold R., Pacheu-Grau D., Lorenzi I. (2012). MITRAC links mitochondrial protein translocation to respiratory-chain assembly and translational regulation. Cell.

[65] Mashkevich G., Repetto B., Glerum D.M., Jin C., Tzagoloff A. (1997). SHY1, the yeast homolog of the mammalian SURF-1 gene, encodes a mitochondrial protein required for respiration. J. Biol. Chem..

[66] Formosa L.E., Maghool S., Sharpe A.J., Reljic B., Muellner-Wong L., Stroud D.A. (2022). Mitochondrial COA7 is a heme-binding protein with disulfide reductase activity, which acts in the early stages of complex IV assembly. Proc. Natl. Acad. Sci. U S A..

[67] Obi C.D., Dailey H.A., Jami-Alahmadi Y., Wohlschlegel J.A., Medlock A.E. (2023). Proteomic analysis of ferrochelatase interactome in erythroid and non-erythroid cells. Life (Basel).

[68] Medlock A.E., Shiferaw M.T., Marcero J.R., Vashisht A.A., Wohlschlegel J.A., Phillips J.D. (2015). Identification of the mitochondrial heme metabolism complex. PLoS One.

[69] Arnold P.K., Finley L.W.S. (2023). Regulation and function of the mammalian tricarboxylic acid cycle. J. Biol. Chem..

[70] Burch J.S., Marcero J.R., Maschek J.A., Cox J.E., Jackson L.K., Medlock A.E. (2018). Glutamine via alpha-ketoglutarate dehydrogenase provides succinyl-CoA for heme synthesis during erythropoiesis. Blood.

[71] Furuyama K., Sassa S. (2000). Interaction between succinyl CoA synthetase and the heme-biosynthetic enzyme ALAS-E is disrupted in sideroblastic anemia. J. Clin. Invest..

[72] Bishop D.F., Tchaikovskii V., Hoffbrand A.V., Fraser M.E., Margolis S. (2012). X-linked sideroblastic anemia due to carboxyl-terminal ALAS2 mutations that cause loss of binding to the beta-subunit of succinyl-CoA synthetase (SUCLA2). J. Biol. Chem..

[73] Kubota Y., Nomura K., Katoh Y., Yamashita R., Kaneko K., Furuyama K. (2016). Novel mechanisms for heme-dependent degradation of ALAS1 protein as a component of negative feedback regulation of heme biosynthesis. J. Biol. Chem..

[74] Lathrop J.T., Timko M.P. (1993). Regulation by heme of mitochondrial protein transport through a conserved amino acid motif. Science.

[75] Chitrakar I., Roberson A.B., Ayres-Galhardo P.H., Brown B.L. (2026). A reversible feedback mechanism regulating mitochondrial heme synthesis. J. Biol. Chem..

[76] Jayaram D.T., Sivaram P., Biswas P., Dai Y., Sweeny E.A., Stuehr D.J. (2025). Heme allocation in eukaryotic cells relies on mitochondrial heme export through FLVCR1b to cytosolic GAPDH. Nat. Commun..

[77] Mercurio S., Petrillo S., Chiabrando D., Bassi Z.I., Gays D., Camporeale A. (2015). The heme exporter Flvcr1 regulates expansion and differentiation of committed erythroid progenitors by controlling intracellular heme accumulation. Haematologica.

[78] Chiabrando D., Marro S., Mercurio S., Giorgi C., Petrillo S., Vinchi F. (2012). The mitochondrial heme exporter FLVCR1b mediates erythroid differentiation. J. Clin. Invest..

[79] Sun F., Zhao Z., Willoughby M.M., Shen S., Zhou Y., Shao Y. (2022). HRG-9 homologues regulate haem trafficking from haem-enriched compartments. Nature.

[80] Han S., Guo K., Wang W., Tao Y.J., Gao H. (2023). Bacterial TANGO2 homologs are heme-trafficking proteins that facilitate biosynthesis of cytochromes c. mBio.

[81] Sweeny E.A., Singh A.B., Chakravarti R., Martinez-Guzman O., Saini A., Haque M.M., Garee G. (2018). Glyceraldehyde-3-phosphate dehydrogenase is a chaperone that allocates labile heme in cells. J. Biol. Chem..

[82] Morishima Y., Lau M., Pratt W.B., Osawa Y. (2023). Dynamic cycling with a unique Hsp90/Hsp70-dependent chaperone machinery and GAPDH is needed for heme insertion and activation of neuronal NO synthase. J. Biol. Chem..

[83] Islam S., Jayaram D.T., Biswas P., Stuehr D.J. (2024). Functional maturation of cytochromes P450 3A4 and 2D6 relies on GAPDH- and Hsp90-Dependent heme allocation. J. Biol. Chem..

[84] Dai Y., Sweeny E.A., Schlanger S., Ghosh A., Stuehr D.J. (2020). GAPDH delivers heme to soluble guanylyl cyclase. J. Biol. Chem..

[85] Tupta B., Stuehr E., Sumi M.P., Sweeny E.A., Smith B., Stuehr D.J. (2022). GAPDH is involved in the heme-maturation of myoglobin and hemoglobin. FASEB J..

[86] Biswas P., Dai Y., Stuehr D.J. (2022). Indoleamine dioxygenase and tryptophan dioxygenase activities are regulated through GAPDH- and Hsp90-dependent control of their heme levels. Free Radic. Biol. Med..

[87] Stuehr D.J., Dai Y., Biswas P., Sweeny E.A., Ghosh A. (2022). New roles for GAPDH, Hsp90, and NO in regulating heme allocation and hemeprotein function in mammals. Biol. Chem..

[88] Hanna D.A., Harvey R.M., Martinez-Guzman O., Yuan X., Chandrasekharan B., Raju G. (2016). Heme dynamics and trafficking factors revealed by genetically encoded fluorescent heme sensors. Proc. Natl. Acad. Sci. U. S. A..

[89] Sandkuhler S.E., Youngs K.S., Gottipalli O., Owlett L.D., Bandora M.B., Naaz A. (2026). Cross-species evaluation of TANGO2 homologs, including HRG-9 and HRG-10 in Caenorhabditis elegans, challenges a proposed role in heme trafficking. Elife.

[90] Kenny T.C., Khan A., Son Y., Yue L., Heissel S., Sharma A. (2023). Integrative genetic analysis identifies FLVCR1 as a plasma-membrane choline transporter in mammals. Cell Metab..

[91] Son Y., Kenny T.C., Khan A., Birsoy K., Hite R.K. (2024). Structural basis of lipid head group entry to the Kennedy pathway by FLVCR1. Nature.

[92] Ri K., Weng T.H., Claveras Cabezudo A., Josting W., Zhang Y. (2024). Molecular mechanism of choline and ethanolamine transport in humans. Nature.

[93] Cater R.J., Mukherjee D., Gil-Iturbe E., Erramilli S.K., Chen T., Koo K. (2024). Structural and molecular basis of choline uptake into the brain by FLVCR2. Nature.

[94] Stentenbach M., Hughes L.A., Fagan S.V., Payne B., Rudler D.L., Siira S.J. (2025). TANGO2 binds crystallin alpha B and its loss causes desminopathy. Nat. Commun..

[95] Lujan A.L., Foresti O., Wojnacki J., Bigliani G., Brouwers N., Pena M.J. (2025). TANGO2 is an acyl-CoA binding protein. J. Cell Biol..

[96] Li H., Xu Z., Guo J., Zhang P., Dong X., Zhao L. (2025). Vitamin B5 monotherapy improves symptoms in a 7-Year-Old girl with TANGO2 deficiency disorder. Am. J. Med. Genet. A.

[97] Piel R.B., Shiferaw M.T., Vashisht A.A., Marcero J.R., Praissman J.L., Phillips J.D. (2016). A novel role for progesterone receptor membrane component 1 (PGRMC1): a partner and regulator of ferrochelatase. Biochemistry.

[98] Piel iii R.B., Obi CD, Viana M.P., Willoughby M.M., Martinez-Guzman O., Wadley A. (2026). Progesterone receptor membrane component 1 (PGRMC1) regulates Heme trafficking through mitochondria-ER junctions. J. Inorg. Biochem.

[99] Galmozzi A., Kok B.P., Kim A.S., Montenegro-Burke J.R., Lee J.Y., Spreafico R. (2019). PGRMC2 is an intracellular haem chaperone critical for adipocyte function. Nature.

[100] Costa-Mattioli M., Walter P. (2020). The integrated stress response: from mechanism to disease. Science.

[101] Anand R., Wai T., Baker M.J., Kladt N., Schauss A.C., Rugarli E (2014). The i-AAA protease YME1L and OMA1 cleave OPA1 to balance mitochondrial fusion and fission. J. Cell Biol..

[102] Rainbolt T.K., Lebeau J., Puchades C., Wiseman R.L (2016). Reciprocal degradation of YME1L and OMA1 adapts mitochondrial proteolytic activity during stress. Cell Rep..

[103] Sekine Y., Houston R., Eckl E.M., Fessler E., Narendra D.P., Jae L.T. (2023). A mitochondrial iron-responsive pathway regulated by DELE1. Mol. Cell.

[104] Kanta S., Vinciauskaite V., Neill G., Muqit M.M K., Masson G.R (2025). Structural insights into allosteric inhibition of HRI kinase by heme binding via HDX-MS. Biochem. J..

[105] Rafie-Kolpin M., Chefalo P.J., Hussain Z., Hahn J., Uma S., Matts R.L (2000). Two heme-binding domains of heme-regulated eukaryotic initiation factor-2alpha kinase. N terminus and kinase insertion. J. Biol. Chem..

[106] Harding H.P., Zhang Y., Zeng H., Novoa I., Lu P.D., Calfon M. (2003). An integrated stress response regulates amino acid metabolism and resistance to oxidative stress. Mol. Cell.

[107] Yu Y., Radisky E., Leibold E.A. (1992). The iron-responsive element binding protein. Purification, cloning, and regulation in rat liver. J. Biol. Chem..

[108] Hirling H., Henderson B.R., Kuhn L.C. (1994). Mutational analysis of the [4Fe-4S]-cluster converting iron regulatory factor from its RNA-binding form to cytoplasmic aconitase. EMBO J..

[109] Gardner P.R., Raineri I., Epstein L.B., White C.W (1995). Superoxide radical and iron modulate aconitase activity in mammalian cells. J. Biol. Chem..

[110] Meyron-Holtz E.G., Ghosh M.C., Iwai K., LaVaute T., Brazzolotto X., Berger U.V (2004). Genetic ablations of iron regulatory proteins 1 and 2 reveal why iron regulatory protein 2 dominates iron homeostasis. EMBO J..

[111] Yamanaka K., Ishikawa H., Megumi Y., Tokunaga F., Kanie M., Rouault T.A (2003). Identification of the ubiquitin-protein ligase that recognizes oxidized IRP2. Nat. Cell Biol..

[112] Ishikawa H., Kato M., Hori H., Ishimori K., Kirisako T., Tokunaga F. (2005). Involvement of heme regulatory motif in heme-mediated ubiquitination and degradation of IRP2. Mol. Cell.

[113] Nishitani Y., Okutani H., Takeda Y., Uchida T., Iwai K., Ishimori K (2019). Specific heme binding to heme regulatory motifs in iron regulatory proteins and its functional significance. J. Inorg. Biochem..

[114] Schranzhofer M., Schifrer M., Cabrera J.A., Kopp S., Chiba P., Beug H. (2006). Remodeling the regulation of iron metabolism during erythroid differentiation to ensure efficient heme biosynthesis. Blood.

[115] Chen J.J., Zhang S. (2019). Heme-regulated eIF2alpha kinase in erythropoiesis and hemoglobinopathies. Blood.

[116] Suzuki H., Tashiro S., Hira S., Sun J., Yamazaki C., Zenke Y. (2004). Heme regulates gene expression by triggering Crm1-dependent nuclear export of Bach1. EMBO J..

[117] Reichard J.F., Motz G.T., Puga A. (2007). Heme oxygenase-1 induction by NRF2 requires inactivation of the transcriptional repressor BACH1. Nucleic Acids Res..

[118] Alam J., Stewart D., Touchard C., Boinapally S., Choi A.M., Cook J.L. (1999). Nrf2, a Cap’n’Collar transcription factor, regulates induction of the heme oxygenase-1 gene. J. Biol. Chem..

[119] Sun J., Hoshino H., Takaku K., Nakajima O., Muto A., Suzuki H. (2002). Hemoprotein Bach1 regulates enhancer availability of heme oxygenase-1 gene. EMBO J..

[120] Sun J., Brand M., Zenke Y., Tashiro S., Groudine M., Igarashi K. (2004). Heme regulates the dynamic exchange of Bach1 and NF-E2-related factors in the Maf transcription factor network. Proc. Natl. Acad. Sci. U. S. A..

[121] Wu N., Yin L., Hanniman E.A., Joshi S., Lazar M.A (2009). Negative feedback maintenance of heme homeostasis by its receptor. Rev-erbalpha, Genes Dev..

[122] Carter E.L., Gupta N., Ragsdale S.W. (2016). High affinity heme binding to a heme regulatory motif on the nuclear receptor rev-erbbeta leads to its degradation and indirectly regulates its interaction with nuclear receptor corepressor. J. Biol. Chem..

[123] Carter E.L., Ramirez Y., Ragsdale S.W. (2017). The heme-regulatory motif of nuclear receptor Rev-erbbeta is a key mediator of heme and redox signaling in circadian rhythm maintenance and metabolism. J. Biol. Chem..

[124] Sarkar A., Carter E.L., Harland J.B., Speelman A.L., Lehnert N., Ragsdale S.W (2021). Ferric heme as a CO/NO sensor in the nuclear receptor rev-Erbss by coupling gas binding to electron transfer. Proc. Natl. Acad. Sci. U S A..

[125] Crumbley C., Wang Y., Kojetin D.J., Burris T.P (2010). Characterization of the core mammalian clock component, NPAS2, as a REV-ERBalpha/RORalpha target gene. J. Biol. Chem..

[126] Tackenberg M.C., Heliodoro K.M., Melink L.C., Liu Y., Zhu K., Lazar M.A (2025). Normal circadian period length requires repression of Npas2 by REV-ERB nuclear receptors. Cell Rep..

[127] Ferreira R., Ohneda K., Yamamoto M., Philipsen S (2005). GATA1 function, a paradigm for transcription factors in hematopoiesis. Mol. Cell Biol..

[128] Fujiwara T., O’Geen H., Keles S., Blahnik K., Linnemann A.K., Kang Y.A. (2009). Discovering hematopoietic mechanisms through genome-wide analysis of GATA factor chromatin occupancy. Mol. Cell.

[129] Welch J.J., Watts J.A., Vakoc C.R., Yao Y., Wang H., Hardison R.C (2004). Global regulation of erythroid gene expression by transcription factor GATA-1. Blood.

[130] Handschin C., Lin J., Rhee J., Peyer A.K., Chin S., Wu P.H. (2005). Nutritional regulation of hepatic heme biosynthesis and porphyria through PGC-1alpha. Cell.

[131] Coste H., Rodriguez J.C. (2002). Orphan nuclear hormone receptor Rev-erbalpha regulates the human apolipoprotein CIII promoter. J Biol Chem.

[132] Raspe E., Duez H., Mansen A., Fontaine C., Fievet C., Fruchart J.C. (2002). Identification of Rev-erbalpha as a physiological repressor of apoC-III gene transcription. J Lipid Res.

[133] Dai Y., Faul E.M., Ghosh A., Stuehr (2022). NO rapidly mobilizes cellular heme to trigger assembly of its own receptor. Proc. Natl. Acad. Sci. U. S. A..

[134] Stuehr D.J., Biswas P., Dai Y., Ghosh A., Islam S., Jayaram D.T. (2023). A natural heme deficiency exists in biology that allows nitric oxide to control heme protein functions by regulating cellular heme distribution. Bioessays.

[135] Ghosh A., Sumi M.P., Tupta B., Okamoto T., Aulak K., Tsutsui M. (2022). Low levels of nitric oxide promotes heme maturation into several hemeproteins and is also therapeutic. Redox Biol..

[136] Faller M., Matsunaga M., Yin S., Loo J.A., Guo F (2007). Heme is involved in microRNA processing. Nat. Struct. Mol. Biol..

[137] Lee S., Lee S., Desnick R., Yasuda M., Lai E.C. (2024). Noncanonical role of ALAS1 as a heme-independent inhibitor of small RNA-mediated silencing. Science.

[138] Puy H., Gouya L., Deybach J.C. (2010). Porphyrias. Lancet.

[139] Phillips J.D. (2019). Heme biosynthesis and the porphyrias. Mol. Genet. Metab..

[140] Bottomley S.S., May B.K., Cox T.C., Cotter P.D., Bishop D.F (1995). Molecular defects of erythroid 5-aminolevulinate synthase in X-linked sideroblastic anemia. J. Bioenerg. Biomembr..

[141] Yoon T., Cowan J.A. (2004). Frataxin-mediated iron delivery to ferrochelatase in the final step of heme biosynthesis. J. Biol. Chem..

[142] Campuzano V., Montermini L., Molto M.D., Pianese L., Cossee M., Cavalcanti F. (1996). Friedreich’s ataxia: autosomal recessive disease caused by an intronic GAA triplet repeat expansion. Science.

[143] Want K., Gorny H., Turki E., Noiray M., Monfort B., Mor-Gautier R (2026). Cross-regulation of [2Fe-2S] cluster synthesis by ferredoxin-2 and frataxin. Nature.

[144] Zhu Z., Yao J., Johns T., Fu K., De Bie I., Macmillan C. (1998). SURF1, encoding a factor involved in the biogenesis of cytochrome c oxidase, is mutated in Leigh syndrome. Nat. Genet..

[145] Tiranti V., Hoertnagel K., Carrozzo R., Galimberti C., Munaro M., Granatiero M (1998). Mutations of SURF-1 in Leigh disease associated with cytochrome c oxidase deficiency. Am. J. Hum. Genet..

[146] Tiranti V., Jaksch M., Hofmann S., Galimberti C., Hoertnagel K., Lulli L. (1999). Loss-of-function mutations of SURF-1 are specifically associated with Leigh syndrome with cytochrome c oxidase deficiency. Ann. Neurol..

[147] Poyau A., Buchet K., Bouzidi M.F., Zabot M.T., Echenne B., Yao J. (2000). Missense mutations in SURF1 associated with deficient cytochrome c oxidase assembly in Leigh syndrome patients. Hum. Genet..

[148] Valnot I., von Kleist-Retzow J.C., Barrientos A., Gorbatyuk M., Taanman J.W., Mehaye B. (2000). A mutation in the human heme a:farnesyltransferase gene (COX10) causes cytochrome c oxidase deficiency. Hum. Mol. Genet..

[149] Antonicka H., Mattman A., Carlson C.G., Glerum D.M., Hoffbuhr K.C., Leary S.C. (2003). Mutations in COX15 produce a defect in the mitochondrial heme biosynthetic pathway, causing early-onset fatal hypertrophic cardiomyopathy. Am. J. Hum. Genet..

[150] Tiwari A., Bahr A., Bahr L., Fleischhauer J., Zinkernagel M.S., Winkler N. (2016). Next generation sequencing based identification of disease-associated mutations in Swiss patients with retinal dystrophies. Sci. Rep..

[151] Yusuf I.H., Shanks M.E., Clouston P., MacLaren R.E (2018). A splice-site variant in FLVCR1 produces retinitis pigmentosa without posterior column ataxia. Ophthalmic Genet..

[152] Rey M.A., Duffy S.P., Brown J.K., Kennedy J.A., Dick J.E., Dror Y. (2008). Enhanced alternative splicing of the FLVCR1 gene in diamond blackfan anemia disrupts FLVCR1 expression and function that are critical for erythropoiesis. Haematologica.

[153] Calame D.G., Wong J.H., Panda P., Nguyen D.T., Leong N.C.P., Sangermano R (2025). Biallelic variation in the choline and ethanolamine transporter FLVCR1 underlies a severe developmental disorder spectrum. Genet. Med..

[154] Puspita L., Chung S.Y., Shim J.W. (2017). Oxidative stress and cellular pathologies in Parkinson's disease. Mol. Brain.

[155] Lin M.T., Beal M.F. (2006). Mitochondrial dysfunction and oxidative stress in neurodegenerative diseases. Nature.

[156] Fiorito V., Allocco A.L., Petrillo S., Gazzano E., Torretta S., Marchi S (2021). The heme synthesis-export system regulates the tricarboxylic acid cycle flux and oxidative phosphorylation. Cell Rep..

[157] Fiorito V., Chiabrando D., Petrillo S., Bertino F., Tolosano E (2019). The multifaceted role of heme in cancer. Front. Oncol..

[158] Destefanis F., Fiorito V., Altruda F., Tolosano E (2019). .Investigating the connection between endogenous heme accumulation and COX2 activity in cancer cells. Front. Oncol..

[159] Lignitto L., LeBoeuf S.E., Homer H., Jiang S., Askenazi M., Karakousi T.R (2019). Nrf2 activation promotes lung cancer metastasis by inhibiting the degradation of Bach1. Cell.

[160] Lee J., Yesilkanal A.E., Wynne J.P., Frankenberger C., Liu J., Yan J (2019). Effective breast cancer combination therapy targeting BACH1 and mitochondrial metabolism. Nature.

[161] Schipper H.M., Cisse S., Stopa E.G. (1995). Expression of heme oxygenase-1 in the senescent and alzheimer-diseased brain. Ann. Neurol..

[162] Smith M.A., Kutty R.K., Richey P.L., Yan S.D., Stern D., Chader G.J. (1994). Heme oxygenase-1 is associated with the neurofibrillary pathology of Alzheimer’s disease. Am. J. Pathol..

[163] Kimpara T., Takeda A., Yamaguchi T., Arai H., Okita N., Takase S. (2000). Increased bilirubins and their derivatives in cerebrospinal fluid in Alzheimer’s disease. Neurobiol. Aging.

[164] Tang X.D., Xu R., Reynolds M.F., Garcia M.L., Heinemann S.H., Hoshi T (2003). Haem can bind to and inhibit mammalian calcium-dependent Slo1 BK channels. Nature.

[165] Tang X.D., Garcia M.L., Heinemann S.H., Hoshi T (2004). Reactive oxygen species impair Slo1 BK channel function by altering cysteine-mediated calcium sensing. Nat. Struct. Mol. Biol..

[166] Sahoo N., Hoshi T., Heinemann S.H. (2014). Oxidative modulation of voltage-gated potassium channels. Antioxid. Redox Signal..

[167] Sahoo N., Goradia N., Ohlenschlager O., Schonherr R., Friedrich M., Plass W (2013). Heme impairs the ball-and-chain inactivation of potassium channels. Proc. Natl. Acad. Sci. U. S. A..

[168] Dietz J.V., Willoughby M.M., Piel R.B., Ross T.A., Bohovych I., Addis H.G. (2021). Mitochondrial contact site and cristae organizing system (MICOS) machinery supports heme biosynthesis by enabling optimal performance of ferrochelatase. Redox Biol..

[169] Aich A., Freundlich M., Vekilov P.G. (2015). The free heme concentration in healthy human erythrocytes. Blood Cells Mol. Dis..

[170] Donegan R.K., Moore C.M., Hanna D.A., Reddi A.R (2019). Handling heme: the mechanisms underlying the movement of heme within and between cells. Free Radic. Biol. Med..

[171] Hopp M.T., Schmalohr B.F., Kuhl T., Detzel M.S., Wissbrock A., Imhof D (2020). Heme determination and quantification methods and their suitability for practical applications and everyday use. Anal. Chem..

